# Genome-Wide Analysis Reveals the Vacuolar pH-Stat of *Saccharomyces cerevisiae*

**DOI:** 10.1371/journal.pone.0017619

**Published:** 2011-03-14

**Authors:** Christopher L. Brett, Laura Kallay, Zhaolin Hua, Richard Green, Anthony Chyou, Yongqiang Zhang, Todd R. Graham, Mark Donowitz, Rajini Rao

**Affiliations:** 1 Department of Physiology, Johns Hopkins University School of Medicine, Baltimore, Maryland, United States of America; 2 Department of Biological Sciences, Vanderbilt University, Nashville, Tennessee, United States of America; 3 Department of Medicine, Johns Hopkins University School of Medicine, Baltimore, Maryland, United States of America; University of Washington, United States of America

## Abstract

Protons, the smallest and most ubiquitous of ions, are central to physiological processes. Transmembrane proton gradients drive ATP synthesis, metabolite transport, receptor recycling and vesicle trafficking, while compartmental pH controls enzyme function. Despite this fundamental importance, the mechanisms underlying pH homeostasis are not entirely accounted for in any organelle or organism. We undertook a genome-wide survey of vacuole pH (pH_v_) in 4,606 single-gene deletion mutants of *Saccharomyces cerevisiae* under control, acid and alkali stress conditions to reveal the vacuolar pH-stat. Median pH_v_ (5.27±0.13) was resistant to acid stress (5.28±0.14) but shifted significantly in response to alkali stress (5.83±0.13). Of 107 mutants that displayed aberrant pH_v_ under more than one external pH condition, functional categories of transporters, membrane biogenesis and trafficking machinery were significantly enriched. Phospholipid flippases, encoded by the family of P4-type ATPases, emerged as pH regulators, as did the yeast ortholog of Niemann Pick Type C protein, implicated in sterol trafficking. An independent genetic screen revealed that correction of pH_v_ dysregulation in a *neo1^ts^* mutant restored viability whereas cholesterol accumulation in human *NPC1^−/−^* fibroblasts diminished upon treatment with a proton ionophore. Furthermore, while it is established that lumenal pH affects trafficking, this study revealed a reciprocal link with many mutants defective in anterograde pathways being hyperacidic and retrograde pathway mutants with alkaline vacuoles. In these and other examples, pH perturbations emerge as a hitherto unrecognized phenotype that may contribute to the cellular basis of disease and offer potential therapeutic intervention through pH modulation.

## Introduction

The ability to control compartmental pH is an essential cellular function. Pioneering work by Metchnikoff and de Duve emphasized the importance of lumenal acidification for bacterial killing in phagosomes, and within lysosomes for acid hydrolase maturation and activity [1], [2]. Additionally, a critical role for lumenal pH within the endocytic pathway has been implicated in the recruitment and assembly of trafficking machinery, reorganization of membrane lipids and actin cytoskeleton in directional vesicle trafficking and in organellar morphology [3], [4], [5], [6]. These cellular pathways contribute to normal cell development and to almost all cell functions from plants to humans. Moreover, defects in pH homeostasis at the organellar level lead to bacterial infection or specific disorders including Dent's disease and numerous lysosomal storage diseases that are linked to pH dysregulation of endosomes and lysosomes, respectively [7], [8], [9], [10].

The H^+^ electrochemical gradient in endosomes and lysosomes is derived from the balance of H^+^ loading and leak across the membrane [11], [12], [13], [14]. The organellar V-type H^+^ ATPase is the major source of lumenal protons [6], [15], [16], [17]. Charge compensation is also critical for the build up of a significant H^+^ chemical gradient (ΔpH_v_), since the V-ATPase is electrogenic, and there is evidence for both inward movement of Cl^−^ ions and outward movement of cations [13]. H^+^ leak pathways modulate lumenal pH and are unmasked when the V-ATPase is blocked. Faster leak rates in endosomes when compared to lysosomes correlates with organelle buffering capacity and surface area [15], [18]. A family of intracellular Na^+^(K^+^)/H^+^ exchangers, represented by mammalian isoforms NHE6-9 with overlapping distribution throughout the endocytic pathway and *trans* Golgi network, are emerging as significant contributors to H^+^ leak [19], [20], [21]. Genetic variants in human NHE6 and NHE9 have been associated with severe X-linked mental retardation, autism, attention deficit hyperactivity disorder and epilepsy [22], [23], [24]. We found that overexpression or deletion of Nhx1, the orthologous Na^+^(K^+^)/H^+^ exchanger in the late endosome of *Saccharomyces cerevisiae*, resulted in alkalinization or acidification of vacuole pH, respectively [20], [25]. However, Nhx1 does not fully account for the leak, and many other contributors to H^+^ leak and lumenal buffering remain to be identified. To find these and other mechanisms of pH regulation, we conducted a genome-wide survey of vacuolar pH using the *S. cerevisiae* library of viable, single gene deletions. We identified 107 gene deletions with significant alterations in vacuole pH under more than one external pH condition. Unexpectedly, mechanisms that control membrane composition such as the P4-ATPase type lipid flippases, components of ergosterol biogenesis and transport including the ortholog of Neimann Pick disease Type C1 protein, were found to be important contributors to vacuole pH homeostasis.

## Results and Discussion

### A genome-wide screen identifies contributors to vacuole pH homeostasis

**Figure 1:**
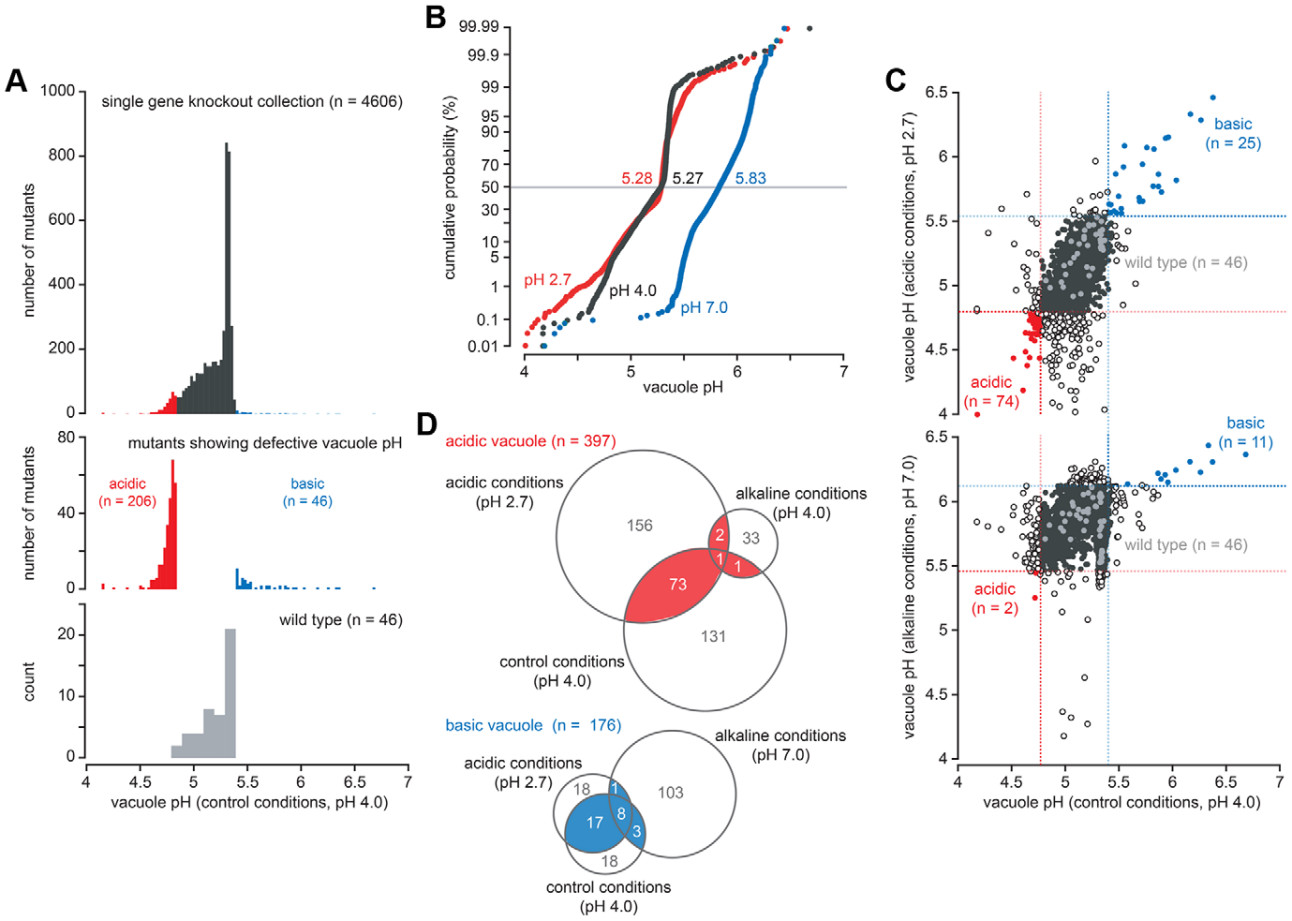
A genome-wide screen for mutations that disrupt vacuole pH in yeast. (a) Distribution of vacuole pH values at external pH 4.0. *Top panel*, a histogram showing the distribution of vacuole pH values observed in the ResGen collection of single gene knockout strains (n = 4606). Outliers (*middle panel*) fall beyond the distribution of vacuole pH values measured in wild type cells (*bottom panel*, n = 46). (b) Cumulative probability plots showing the distribution of vacuole pH values measured in the mutant collection at external pH 2.7 (acidic conditions, n = 4469; red), pH 4.0 (control conditions, n = 4606; black) or pH 7.0 (alkaline conditions, n = 4593; blue). Median values are shown for each population (indicated by the horizontal line). (c) Vacuole pH values from the mutant collection grown under acidic conditions (top) or alkaline conditions (bottom) were compared to values measured under control conditions. Minimum (red lines) and maximum (blue lines) pH values observed in the wild type population (light grey points) are indicated for each condition. Mutants with abnormally acidic (red) or alkaline (blue) vacuole under both conditions are highlighted; black closed circles indicate mutants with normal vacuole pH values, black open circles are mutants with abnormal vacuole pH values observed under a single condition. (d) Venn diagram displaying the distribution of outliers identified under all three conditions. ∼20% of the mutants displayed either hyperacidic vacuoles (red, n = 77) or alkaline vacuoles (blue; n = 27) in at least two growth conditions with different pH; these are listed in Table S3.

To gain a comprehensive view of the vacuolar ‘pH-stat’, we measured vacuole pH in a collection of 4,606 viable, single gene deletion strains that span the *S. cerevisiae* genome (Figure 1a; see Supplemental Materials Table S1 for the complete dataset). The majority of these mutant strains (4,336) had vacuole pH values within the range observed in wild type cells (4.81<pH_v_<5.41; Figure 1a, bottom panel), most falling near the median of 5.27±0.13. However, single gene mutants with abnormally acidic (224) or alkaline (46) vacuoles were discovered (Figure 1a, middle panel). Lumenal pH in these mutants reached as low as 4.17 and high as 6.68, representing a sizeable range tolerated by the cell without notable effects on mid-log growth or viability (see Supplemental Materials Figure S1).

We repeated this survey under acidic (pH 2.7) or alkaline (pH 7.0) conditions to unmask mechanisms important for homeostasis in response to pH stress (Figure 1b; see Supplemental Materials Table S1 for the complete dataset). When acid stressed, the median pH_v_ of wild type cells and the mutant population remained the same (5.28±0.14) and a similar number of mutants with acidic vacuoles were observed (206, of which 74 were also acidic under control conditions). However pH_v_ of these mutants strayed further from the median compared to control conditions, consistent with a greater vacuolar accumulation of H^+^ in acidic medium. This is most apparent in Figure 1c, which compares pH_v_ obtained under control conditions against those measured under acid or alkali stress for each mutant strain. In contrast to acid stress, the median pH_v_ of wild type and mutant population jumped from 5.27±0.13 to 5.83±0.13 upon alkali stress. Few mutants with acidic vacuoles were observed under alkali stress (36), and nearly all mutants (with the exception of 4 strains) with hyperacidic vacuoles at external pH≤4.0 were able to recover when external pH was raised. As expected, we observed many more mutants with alkaline vacuoles under this condition (115, of which 12 were also alkaline at lower external pH), consistent with difficulty in scavenging protons when the external H^+^ concentration is low. Notably, the range of vacuolar pH values observed in the mutant population was the same under each condition. Mutants that displayed acidic vacuoles (77) and alkali vacuoles (29) in more than one external pH condition (Figure 1d) are listed in Table S3. Of these 107 genes, three functional categories were significantly enriched: transporters, membrane organization and biogenesis, and membrane trafficking machinery.

### P4-ATPase phospholipid translocases regulate vacuole pH homeostasis

**Figure 2:**
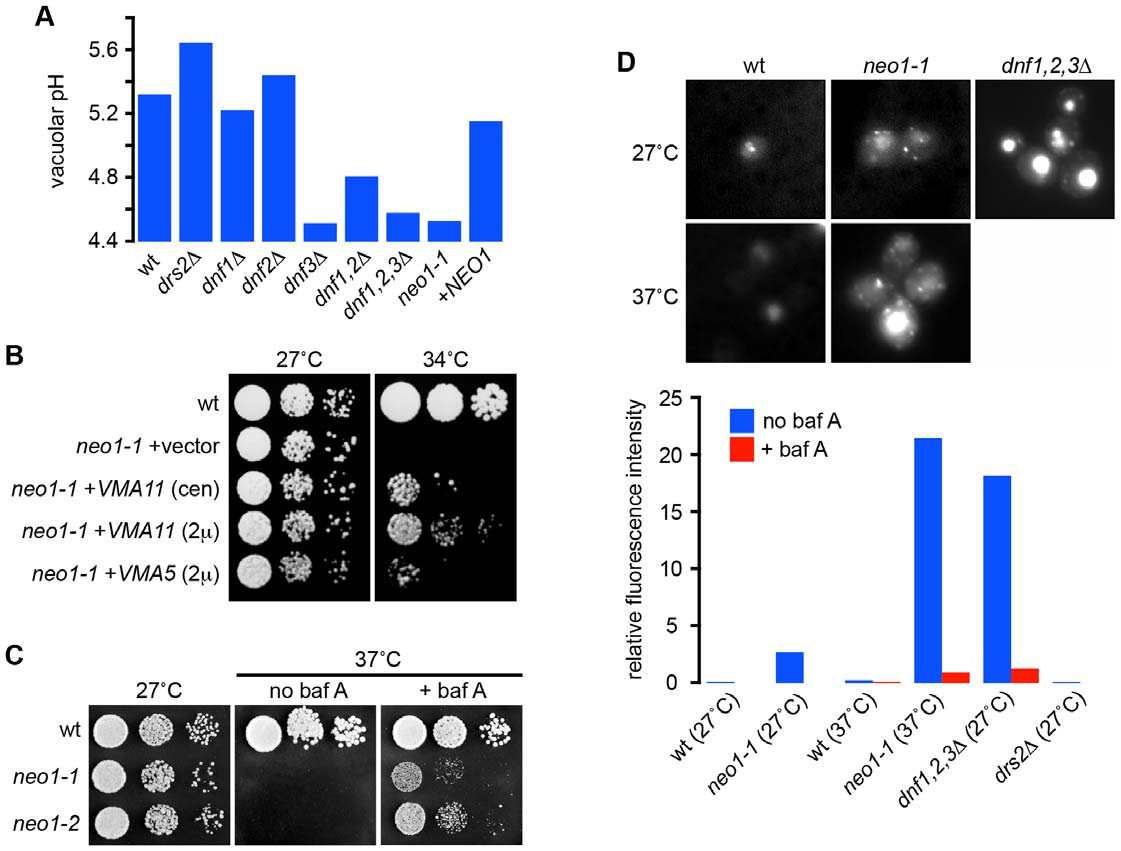
P4-ATPase flippases contribute to vacuole pH regulation. (a) Vacuolar pH is defective in P4-ATPase mutants. Vacuolar pH was measured in wild type BY4742 cells, strains lacking one or more of the P4-ATPases (*drs2Δ*, *dnf1Δ*, *dnf2Δ*, *dnf3Δ*) or with a temperature sensitive allele of the essential *NEO1* gene (*neo1-1*) without or with (+*NEO1*) a plasmid containing wild type *NEO1*. The *neo1* strains were shifted to the nonpermisive temperature for 1 hour (34°C). Data represent average of duplicates and one of three independent experiments. (b) Overexpression of *VMA* genes suppresses the *neo1-1* mutant growth defect. The *neo1-1* strain was transformed with an empty vector, a single copy *VMA11*, a multi-copy *VMA11*, or multi-copy *VMA5* plasmids. Serial dilutions of the transformants were spotted on YPD plates and tested at 27°C and 34°C. (c) Inhibition of V-ATPase function suppresses *neo1-ts* growth defect at nonpermissive temperature. Serial dilution of wild type or two *neo1-ts* mutant cultures were tested on YPD plates containing 5 µM bafilomycin A1. (d) Compartmental hyperacidification in *neo1-1* or triple *dnf1,2,3Δ* mutants. Micrographs show quinacrine staining of wild type (wt), *neo1-1* or *dnf1,2,3Δ* triple mutants at permissive (27°C) and nonpermissive (37°C) temperatures. Fluorescence intensity (a.u., arbitrary units) was quantified by flow cytometry in the absence or presence of 5 µM bafilomycin A1 (baf A). Negative controls include *drs2Δ* cells. 30,000 cells were analyzed for each strain.

Gene disruption of 11 subunits or assembly factors of the vacuolar H^+^-ATPase [6], [15], [16], [17] resulted in prominent vacuole alkalinization (Tables S2 and S3), validating our screen. Only one other member of the transport ATPase category, *drs2Δ*, exhibited a *vma*-like shift in vacuole pH (Table S2; Figure 2a). Drs2 is one of five ATP-driven phospholipid flippases (P4-ATPases) in yeast that contribute to bilayer asymmetry, including Dnf1-3 and Neo1 [26], [27], [28]. Disruption of Dnf3, another member of this group, resulted in an acidic shift. A systematic evaluation of pH_v_ in P4-ATPase mutants (Figure 2a) revealed that *dnf1dnf2Δ* double and *dnf1dnf2dnf3Δ* triple mutants, lacking functionally redundant members of the P4-ATPases, all displayed hyperacidic vacuoles (Figure 2a). In addition, acidic vacuoles were observed in a temperature-sensitive mutant (*neo1-1*) of *NEO1*, the only essential gene of the P4-ATPase family [29]. Furthermore, the acidic shift in pH_v_ was increased in the *neo1-1* mutant following transfer to the non-permissive growth temperature (Figure 2a, d).

Underscoring the functional significance of pH dysregulation in the flippase mutants, an unbiased screen for multicopy suppressors of the *neo1-ts* temperature sensitive growth defect identified *VMA5* and *VMA11*, encoding subunits of the V-ATPase (Figure 2b). Previous work had shown that non-stoichiometric expression of individual VMA subunits disrupted V-ATPase assembly [30], suggesting that reduced V-ATPase activity was responsible for *neo1-ts* suppression. Indeed, disruption of *VMA* genes or addition of the V-ATPase inhibitor bafilomycin A to the growth medium also suppressed the *neo1-1* growth defect (Figure 2c). Relative to wild-type cells, *neo1-ts* and *dnf1,2,3Δ* vacuoles stained brightly with quinacrine, a fluorescent compound that accumulates in acidic compartments, and this phenotype was reversed by bafilomycin A. Quinacrine staining also revealed numerous small puncta in the *neo1-1* mutant in addition to vacuoles (Figure 2d), suggesting that hyperacidification of endosomes and/or Golgi contributes to the growth defect in this mutant.

The opposing effects of different members of the flippase family on vacuole pH may arise from differences in substrate specificity and/or cellular location. In support of this hypothesis, a mutant defective in the biosynthesis of phospholipid substrates of Dnf1-3 *muq1Δ*, also exhibited hyperacidic vacuoles (Table S3) [31], [32]. Drs2 mutants have a unique defect in sterol trafficking [33] and our analysis also revealed the alkalinizing effect of *erg* mutants defective in ergosterol biogenesis (Table S2). Recently, a network of kinases that regulate flippase activity has been identified, with Ypk1 exerting negative regulation on Fpk1, an activator (together with the Fpk2 homolog), of inward translocation of phospholipids by Dnf1 and Dnf2 [31], [34]. The observation that *ypk1Δ* mutants display alkaline pH_v_ (Table S3), suggests that increased activity of the flippases has the opposite effect on pH_v_ compared to the *dnf1,2Δ* deletion. Defects in membrane asymmetry or protein trafficking observed in the P4-ATPase mutants may alter the vacuole membrane thereby perturbing pH_v_ homeostasis, although we cannot exclude that co- or counter-transport of H^+^ accompanies transbilayer movement of lipid headgroups. Thus, our analysis revealed an unexpected role for the P4-ATPase flippase family and transbilayer distribution of phospholipid substrates in vacuole pH homeostasis.

In addition to ATP-driven pumps, we identified transporters of weak acids/bases, H^+^ leak pathways (H^+^ cotransporters and antiporters) and membrane potential shunts (K^+^, Cl^−^ transporters) [13], [35], [36]. Tables S2 and S3 list some examples: Pdr12, a member of the ABC type drug pumps, that has been shown to efflux weak acids at the plasma membrane [37], Nhx1, the endosomal cation/H^+^ antiporter of the NHE superfamily [20], [25], and Mep3, a vacuolar ammonium transporter related to human Rhesus blood group factor [38]. Other transporters listed have not previously been implicated in pH regulation. Therefore their potential ability to translocate H^+^ warrants further investigation. However, in some cases, effects of a transported substrate, such as glucose (e.g., Hxt10) [39] on downstream metabolic pathways could indirectly influence vacuole pH.

### Sterol biogenesis and transport is linked to vacuole pH

**Figure 3:**
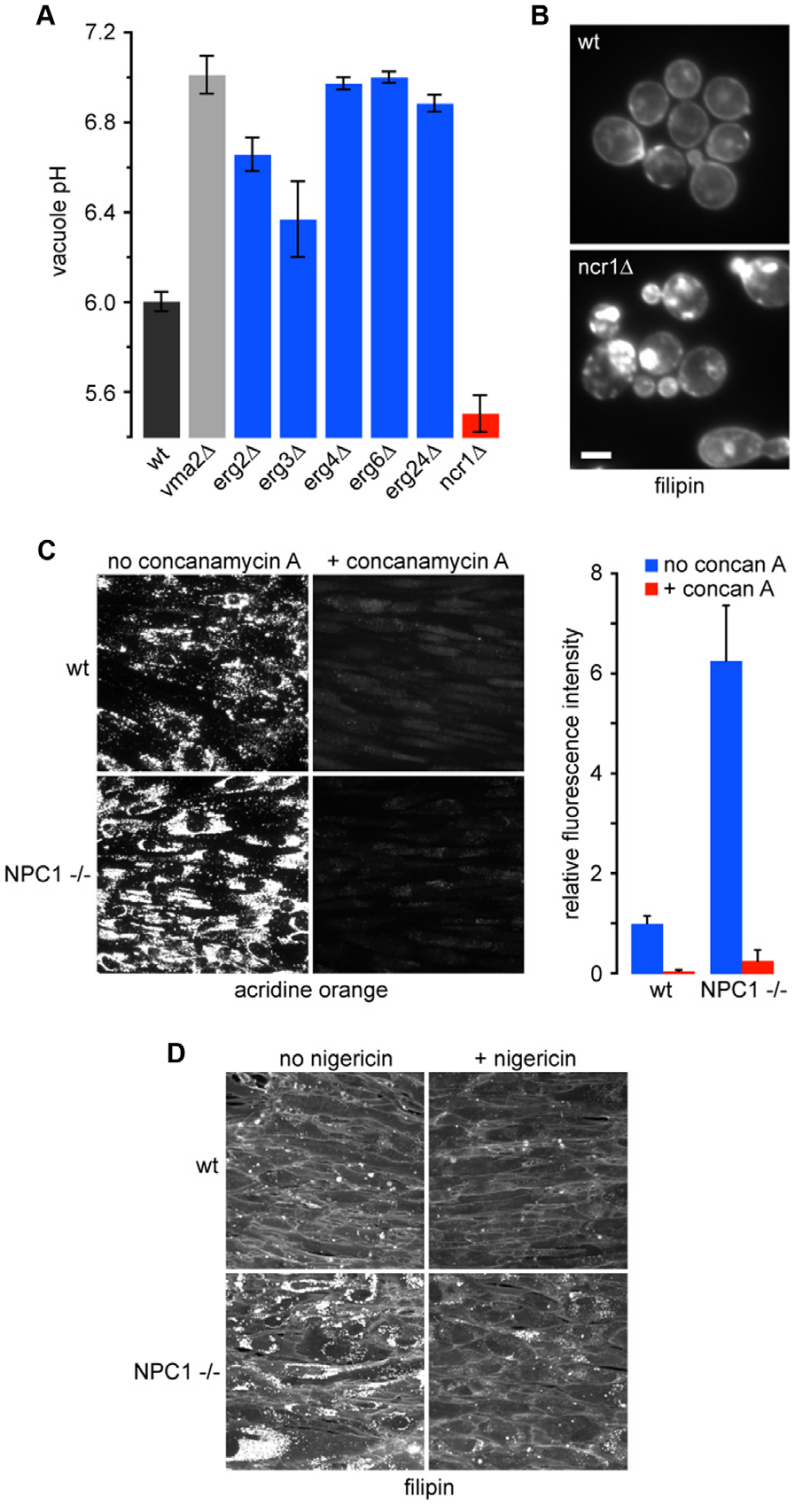
Lysosomal hyperacidification accompanies a cellular Niemann-Pick type C phenotype. (a) Vacuoles in ergosterol accumulating *ncr1Δ* cells are hyperacidic, whereas *erg* mutants lacking ergosterol have abnormally alkaline vacuoles. (b) Intracellular sterol accumulates in *ncr1Δ* yeast. Yeast cells were stained with filipin to detect sterol in the presence or absence of *NCR1*. Bar, 2 µm. (c) Fibroblasts harboring pathogenic alleles of NPC1 (P237S/I1061T) have hyperacidified lysosomes. Wild type cells from a control subject (wt) or mutant cells from a patient with Niemann-Pick type C disease (NPC1^−/−^) were stained with acridine orange in the absence or presence of 10 nM concanamycin A1 (concan A), an inhibitor of the V-ATPase. Bar, 10 µm. Cellular acridine orange fluorescence was quantified relative to untreated wild type cells; bars represent standard deviations (n = 100 cells). (d) Nigericin prevents aberrant accumulation of cholesterol in lysosomes of NPC1^−/−^ mutant cells. Cells were stained with filipin to detect cholesterol in the presence or absence of 10 nM nigericin, a K^+^/H^+^ ionophore.

In addition to phospholipids, sterols are critical components of the lipid bilayer that may impact electrochemical ion gradients by influencing membrane permeability or by modulating the activity of membrane transport proteins. We recently demonstrated a requirement for ergosterol in H^+^ pumping and ATP hydrolysis activity of the V-ATPase [40]. Consistent with this functional requirement, we observed prominent vacuolar alkalization in multiple *erg* mutants defective in sterol biogenesis, including *erg2Δ*, *erg3Δ*, *erg4Δ*, *erg6Δ* and *erg24Δ* (Table S2 and Figure 3a). Conversely, we found a reciprocal acidic pH_v_ shift in *ncr1Δ* strain that accumulates sterol in endosomal compartments, which we demonstrate by intracellular filipin staining (Table S2; Figure 3a) [41], [42]. Similarly, *ste20Δ* mutants, lacking a PAK family kinase that negatively regulates sterol biogenesis, also exhibited increased sterol levels [43] and corresponding acidic shift in pH_v_ (Table S3).

Since Ncr1 is the yeast ortholog of Niemann-Pick type C protein, we sought to evaluate whether the pH perturbations revealed by our analysis represent a new phenotype that may contribute to the cellular basis of disease. As previously reported [44], human NPC1^−/−^ fibroblasts, carrying mutant alleles P237S/I1061T, showed excessive intracellular accumulation of unesterified cholesterol, as revealed by filipin staining, consistent with disruption of cholesterol trafficking in patients with Niemann-Pick Type C disease (Figure 3d). We found that lysosomes of mutant fibroblasts accumulated significantly more acridine orange fluorescence than control, suggesting a shift to hyperacidic pH_v_, as seen in the yeast *ncr1Δ* mutant (Figure 3c). Inhibition of the V-ATPase with concanamycin A eliminated pH-dependent acridine orange fluorescence in both mutant and wild type lysosomes, as expected (Figure 3c). Although intracellular cholesterol accumulation was not abolished by concanamycin A treatment, we noted altered distribution of cholesterol accumulating endosomes to the cell periphery, highlighting the importance of luminal acidification in endocytosis and vesicular trafficking. Therefore, we treated cells with low concentrations of the K^+^/H^+^ ionophore nigericin, sufficient only for transient alkalization of lysosomes. We show that low levels of nigericin drastically reduced accumulation of unesterified cholesterol in the mutant cells, as revealed by filipin staining (Figure 3d), suggesting that a more subtle modulation of lumenal pH and cation homeostasis may be effective in reversing sterol trafficking defects. Reversal of sterol accumulation in late endosomal/llysosomal compartments with a cholesterol extracting agent such as cyclodextrin was sufficient to alleviate liver dysfunction and neurodegeneration in a mouse model of NPC disease [45]. Thus, hyperacidification of organelles may contribute to Niemann-Pick type C disease pathogenesis and compartmental pH modulation may offer therapeutic possibilities in patients with this disease.

### A reciprocal relationship between vesicle trafficking defects and vacuole pH

**Figure 4:**
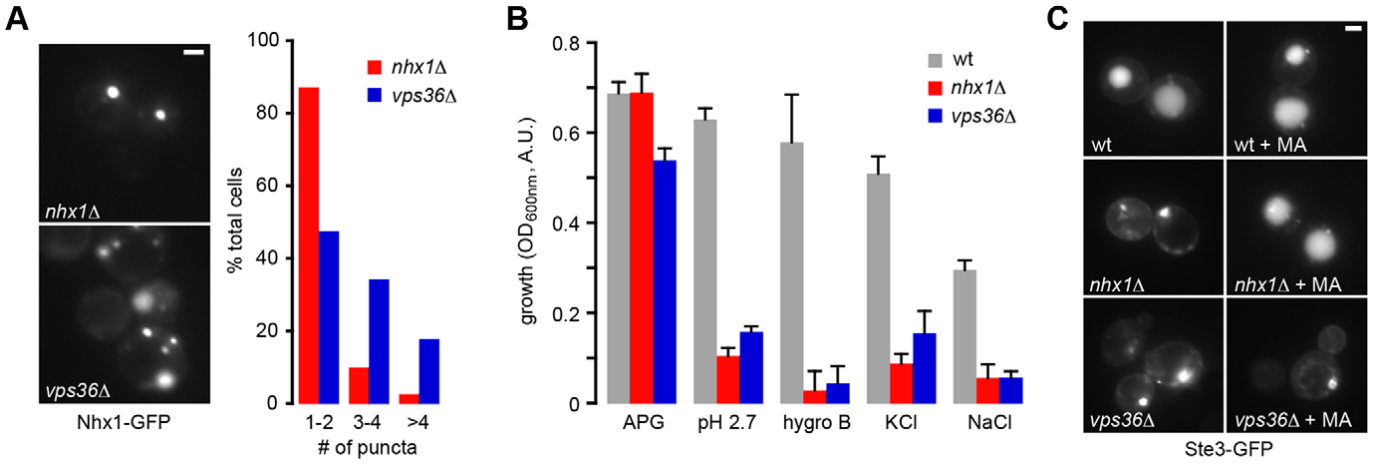
Transporter dysfunction in *vps36*Δ cells. (a) Nhx1 is mislocalized in *vps36*Δ cells. Micrographs show the location of Nhx1-GFP in *nhx1Δ* or *vps36*Δ cells transformed with the 2 µ plasmid pRin82 containing *NHX1:GFP* behind its endogenous promoter. Bar, 2 µm. The number of Nhx1-GFP stained puncta per cell was quantified; >100 cells were analyzed for each strain. (b) *vps36*Δ phenocopies *nhx1*Δ. Growth of wild type BY4742 (wt), *nhx1*Δ or *vps36*Δ cultures was measured after 19 hrs at 30°C under control conditions (APG, pH 4.0), acid stress (pH 2.7), or in the presence of 10 µg/ml hygromycin B (Hygro B), 1.5 M KCl or 1.5 M NaCl. Bars represent mean +/− S.E.M. (c) The pH homeostasis defect in *nhx1*Δ is downstream of Vps36 function. Micrographs show the cellular location of Ste3-GFP in wild type BY4742 (wt), *nhx1*Δ or *vps36*Δ cells grown in APG pH 4.0 medium at 30°C with or without methylamine (MA, 10 mM) treatment. Note that pH amelioration fails to correct trafficking dysfunction in *vps36*Δ cells. Bar, 2 µm.

Biochemical approaches have long established that compartmental trafficking and organelle morphology are pH sensitive [3], [4]. Indeed, screens for mutations that affect vacuole morphology and protein sorting identified known contributors to lumenal pH (e.g. *vma*Δ) [46], [47], [48]. A screen for small molecule inhibitors of endocytic trafficking identified V-ATPase inhibitors [49]. Genetic evidence from our survey revealed a reciprocal link between pH homeostasis and trafficking: 11% of gene disruptions leading to vacuolar pH perturbation are involved in inter-organellar trafficking, with the multivesicular body (MVB) pathway and vacuole fusion machinery being particularly prominent (Table S3; Figure S2). Of note, impairment of retrograde trafficking generally correlated with vacuole alkalinization, whereas defects in anterograde trafficking often led to vacuole hyperacidification (Table S2), although exceptions were observed (e.g., *snf7*Δ/*vps32Δ* displayed alkaline, rather than acidic vacuole). Components of the trafficking machinery may affect compartmental H^+^ concentrations through mislocalization of downstream effectors such as H^+^ transporters and pH regulators. A good example of this is *vps5Δ*, a mutant defective in the late endosome to TGN retrograde pathway, previously shown to missort a TGN Mg^2+^/H^+^ antiporter to the vacuole [50]. Increased cation/H^+^ exchange at the vacuolar membrane would be expected to dissipate the H^+^ gradient by enhancing vacuolar H^+^ leak. In validation of this hypothesis, we observed prominent alkalinization of pH_v_ in *vps5Δ* as well as other mutants lacking components of the retromer complex (*vps5Δ*, *vps17Δ*, *vps29Δ*; Table S3) [51]. Similarly, anterograde trafficking mutants might disrupt localization and function of vacuolar/late endosomal transporters that contribute to the H^+^ leak, with ensuing vacuolar hyperacidification. Previous studies have concluded that localization of Nhx1 is altered in *vps27Δ*, a mutant defective in an early step of the MVB pathway [51], [52], [53]. We asked if other MVB pathway mutants with significantly acidic vacuolar pH shared this phenotype. We observed prominent mislocalization of Nhx1-GFP in *vps36Δ* (Fig. 4a). Furthermore, this mutant was found to phenocopy *nhx1Δ* in growth sensitivity to low pH, hygromycin B and salt stress (Figure 4b) suggesting a tight physiological coupling between transporter function and cellular localization. Addition of the weak base methylamine corrected defective sorting of cargo (Ste3-GFP) in *nhx1Δ*, but not *vps36Δ* (Figure 4c). This clarified that pH dysregulation was the *cause* of the trafficking defect in the transporter mutant, but the *effect* of trafficking dysfunction in the MVB pathway mutant.

In summary, we have identified a genetic pH-stat which functions largely to keep vacuolar pH from becoming hyperacidic or alkaline under metabolic and environmental stress, with many novel contributors being identified. Manipulation of the pH-stat could have industrial applications for bioproduction of proteins or biofuels. Combinatorial deletion (or chemical inhibition) of evolutionarily conserved genes with opposing effects on cellular pH may correct defects in pH regulation, establish causality between pH regulation and cellular functions such as trafficking, and potentially ameliorate disease phenotypes.

## Materials and Methods

### Yeast strains and reagents

For growth and vacuolar pH measurements we used the ResGen library of 4,828 *Saccharomyces cerevisiae* strains with deletion of each nonessential gene in a haploid background (BY4742; Invitrogen, Carlsbad, CA). Mutations of interest were confirmed by PCR (e.g. Supplementary Information Figure S2). Strains carrying mutations in the P4-ATPase genes were constructed in the BY4742/BY4741 background and have been described [29]. All reagents were purchased from Sigma-Aldrich Corp. (St. Louis, MI) or Qbiogene Inc. (Irvine, CA), except 2′,7′-bis(2-carboxyethyl)-5,6-carboxyfluorescein-acetoxymethyl ester (BCECF-AM; Invitrogen Corp., Eugene, OR).

### Measurement of vacuolar pH

Overnight seed cultures of the BY4742 mutant collection (represented in fifty-two 96-well microtiter plates) or selected strains were used to inoculate 200 µl experimental cultures containing APG, a synthetic minimal medium containing 10 mM Arginine, 8 mM Phosphoric acid, 2% Glucose, 2 mM MgSO_4_, 1 mM KCl, 0.2 mM CaCl_2_, and trace minerals and vitamins titrated to pH 4.0 or 2.7 with phosphoric acid or pH 7.0 with KOH and 10 mM MES [20]. On each microtiter plate, two wells were reserved for a wild type (WT) culture and for growth medium alone, representing positive and negative controls respectively. Cultures were grown for 19 h at 30°C and immediately prior to recording growth or pH, cultures were rapidly resuspended using an electromagnetic microtiter plate shaker (Union Scientific, Randallstown, Maryland).

Vacuolar pH measurements were performed using methods previously described with modification to accommodate high-throughput analysis [25]. Briefly, after growth was assessed, experimental cultures were incubated with 50 µM 2′,7′-bis(2-carboxyethyl)-5,6-carboxyfluorescein-acetoxymethyl ester (BCECF-AM; Molecular Probes, Eugene, OR) at 30°C for 30 min., and then washed and resuspended in APG medium at the appropriate pH. Single fluorescence intensity (excitation at 485 nm, emission at 520 nm) and absorbance (at 600 nm) were then measured. Ninety-six independent calibration experiments were performed and vacuolar pH values were calculated as described previously. All fluorescence and absorbance readings were taken at 30°C using a BMG FLUOstar Optima multimode plate reader with accompanying BMG FLUOstar Optima Version 1.20-0 software (BMG Labtechnologies, Durham, NC). Accurate pH estimations could not be made when strains showed severe growth defects (<20% of wild type growth) or poor loading, poor expression or mislocalization of the pH-sensitive probe. Vacuolar pH was not assessed for 6.7% of the mutant collection; vacuolar pH measurements were recorded from 4469, 4606, or 4593 mutants under acid stress, control conditions or alkali stress respectively.

### Data analysis

Mutant strains showing <20% of WT growth under control conditions (pH 4.0) were omitted from analysis. Medians and median absolute deviations (MADs) were calculated for each dataset and descriptive statistical analysis was performed. Mutant strains with vacuolar pH values above the maximum or below the minimum pH_v_ observed in the wild type population (n = 46) were considered to have abnormally alkaline or acidic vacuoles, respectively (Figure 1a; Supplementary Information Table S1). Data were organized, plotted and analyzed using Excel X (Microsoft Corp., Redmond, WA), JMP vs. 5.0.1a (SAS Institute, Carey, NC), and KaliedaGraph vs. 4.0 (Synergy Software Technologies Inc., Essex Junction, VT).

### Isolation of the *neo1-1* temperature sensitive allele and its suppressors

A Yep13 genomic library (ATCC) was used for the *neo1-1* multi-copy suppressor screen. Strain ZHY628-15B (BY4742 *neo1Δ* containing p413-neo1-1) was transformed with library DNA and incubated at 27°C. Transformants were then replicated and incubated at 37°C. Plasmids rescued from suppressor colonies were transformed again into ZHY628-15B and tested for growth at 37°C. A SpeI/SnaBI fragment of one suppressor clone (named pNS7) was inserted into pRS315 or pRS425 to generate the single copy or multi-copy VMA11 plasmids. The plasmid containing VMA5 (Yep352-VMA5) was a gift from Patricia Kane (SUNY Health Sciences Center, Syracuse, NY).

### Quinacrine staining

For quinacrine staining, strains BY4741 (WT), ZHY124-15B1B (*neo1-ts*), PFY3273A (*dnf1,2,3Δ*), ZHY615D1C (*drs2Δ*) were grown to mid-logarithmic phase in YPD at 27°C and half of the *neo1-ts* strain was shifted to 37°C for 2 hrs. Each culture was treated with or without bafilomycin A (5 µM) for 25 minutes prior to harvesting. Cells were resuspended in YPD medium containing 100 mM Hepes pH 7.6, 200 µM quinacrine, 25 µg/ml propidium iodide with or without 5 µM bafilomycin A, and stained for 8 min at 27 or 37°C as indicated in Figure 2d. Stained cells were washed three times with 100 mM Hepes pH 7.6, 0.2% glucose and stored on ice prior measuring the fluorescence intensity of 30,000 living cells using the FITC channel of a fluorescence activated cell sorter. The fluorescence intensity of mock-treated cells incubated without quinacrine were used to subtract background fluorescence. Images of stained cells were captured using a Zeiss Axioplan fluorescence microscope, a cooled CCD camera and MetaMorph 4.5 software (Molecular Devices, Sunnyvale, CA).

### Mammalian cell culture and microscopy

Wild type (GM05659, designated wt) and Niemann-Pick Type C1 (GM03123, designated NPC1^−/−^, a gift from Dr. Laura Liscum, Tufts University School of Medicine) [54] fibroblasts were cultured according to Coriell Cell Repositories recommendations: minimal essential media supplemented with 10% Fetal Bovine Serum, nonessential amino acids and penicillin/streptomycin at 37°C, 5% CO_2_ in a humidified incubator. Cells were plated onto glass coverslips 24 hours prior to treatment to allow adherence then grown an additional 48 hours in the presence of either DMSO, 10 nM Concanamycin A, 10 nM Nigericin or left untreated. Cells were incubated with 1 µM acridine orange in standard bath solution for 10 min at 37°C [54]. Images were acquired with the same parameters (exposure time and fluorescence intensity) using a Zeiss Axiovert fluorescent microscope equipped with a Photometrics CoolSnap CCD camera. Average fluorescence of 4 independent fields of wild type and NPC1 cells for each treatment was measured using MetaMorph image analysis software (Molecular Devices Corperation, Downingtown, PA). Filipin staining was performed by exposing paraformaldehyde fixed cells (3% in PBS) to 25 mg/ml filipin for 30 minutes at room temperature as described [55]. Samples were visualized as described above.

## Supporting Information

Figure S1 **Growth of yeast mutant strains does not correlate with pH_v_.** Defects in vacuole pH do not correlate with poor growth. Vacuole pH values shown in Figure 1b n (≥4469) are plotted against yeast culture growth measured under acidic (*top*), standard (*middle*) or alkaline (*bottom panel*) conditions. Resulting log-log plots indicate that vacuole pH and growth are independent variables.
(PDF)

Figure S2 **Vesicle trafficking defects lead to pH_v_ dysregulation.** Mutants with defective pH_v_ also identified by genome-wide screens for endocytic trafficking defects and/or vacuole fusion defects are shown (numbers indicated in parentheses). Arrows indicate directional trafficking pathways between the following organelles: vacuole (Vac), late endosome (LE), intralumenal vesicles of multivesicular bodies (ILV), *trans*-Golgi network (TGN), Golgi apparatus (GA), endoplasmic reticulum (ER), nucleus (Nu), cytoplasmic (Cyto), plasma membrane (PM), and cell wall (CW); the V-ATPase, autophagic process and cytoskeleton are also shown. The mutants identified either showed basic (blue) or acidic vacuole (red). The pH_v_ of mutants shown are listed in Supplementary Table S2.
(PDF)

Table S1 **Complete dataset of yeast vacuole pH and growth measured at three external pH values.** Vacuole pH and culture growth (Absorbance at 600 nm) values for 4,606 yeast single gene deletion strains examined in this study. Prior to recording, cultures were grown for 19 hours in APG media adjusted to pH 2.7, 4.0 or 7.0. Vacuole pH datasets (also plotted in Figure 1) were sorted and strains are shown in order from lowest to highest vacuole pH. In addition to the sample size, median, and MAD, acidic and basic thresholds and lists of outliers are shown for each condition. Growth datasets were grouped and sorted; strains are shown in order from lowest to highest growth measured under control conditions. Unsorted vacuole pH datasets are also provided.
(XLS)

Table S2 **Major functional groups of the vacuolar pH-stat.** Genes identified in the vacuolar pH survey of the yeast single gene deletion library were sorted into functional categories. Major functional groups of membrane phospholipid and sterol distribution, endocytic trafficking and transporters are listed. Growth and vacuole pH values obtained from strains with gene deletions are shown and outlying vacuole pH values are indicated (acidic, red; basic, blue).
(XLS)

Table S3 **Summary of major contributors to vacuolar pH-stat.** Summary of 107 genes identified as contributors to the vacuole pH-stat under more than one external pH condition (see Figure 1d). The genes are sorted by effect of deletion on vacuole pH and by cellular function.
(PDF)
