# Supplementary figures and images for: Genome-Wide Analysis Reveals the Vacuolar pH-Stat of Saccharomyces cerevisiae
Source: PLoS One. 2011 Mar 14;6(3):e17619. doi: 10.1371/journal.pone.0017619 (PMC3056714; doi:10.1371/journal.pone.0017619)

Figure S1

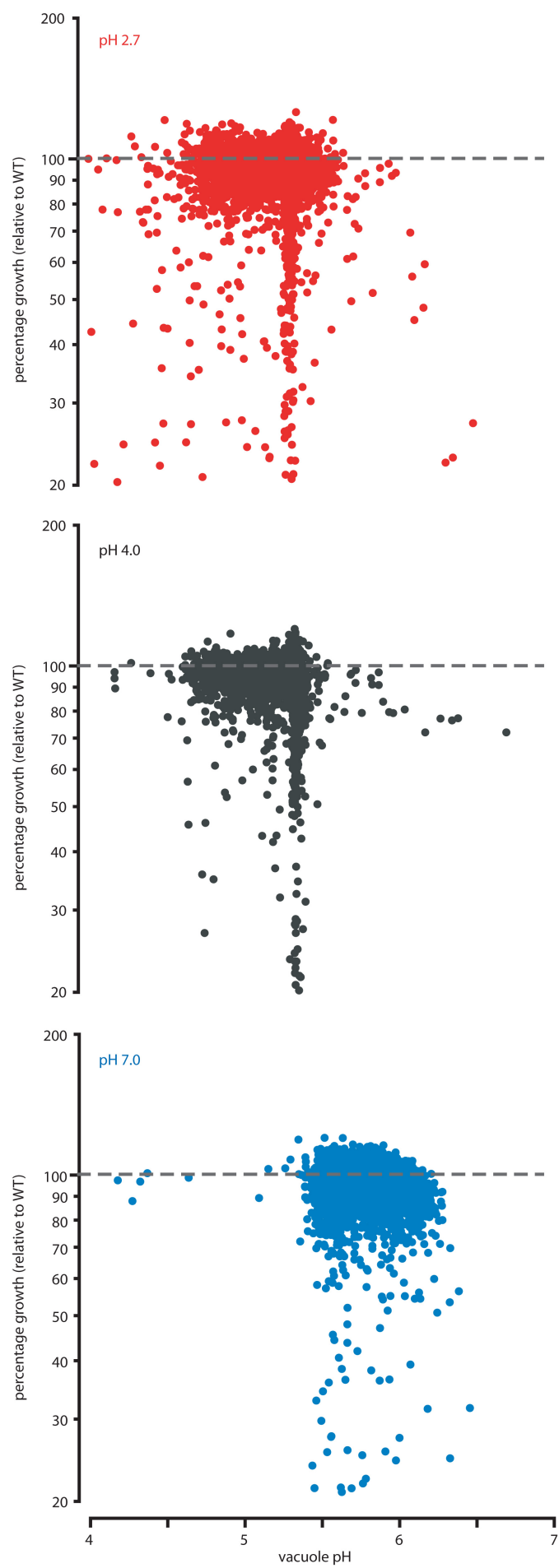

Supplement: Figure S1 — Growth of yeast mutant strains does not correlate with pHv. Defects in vacuole pH do not correlate with poor growth. Vacuole pH values shown in Figure 1b n (≥4469) are plotted against yeast culture growth measured under acidic (top), standard (middle) or alkaline (bottom panel) conditions. Resulting log-log plots indicate that vacuole pH and growth are independent variables. (PDF) [file pone.0017619.s001.pdf]

**Figure S2.**

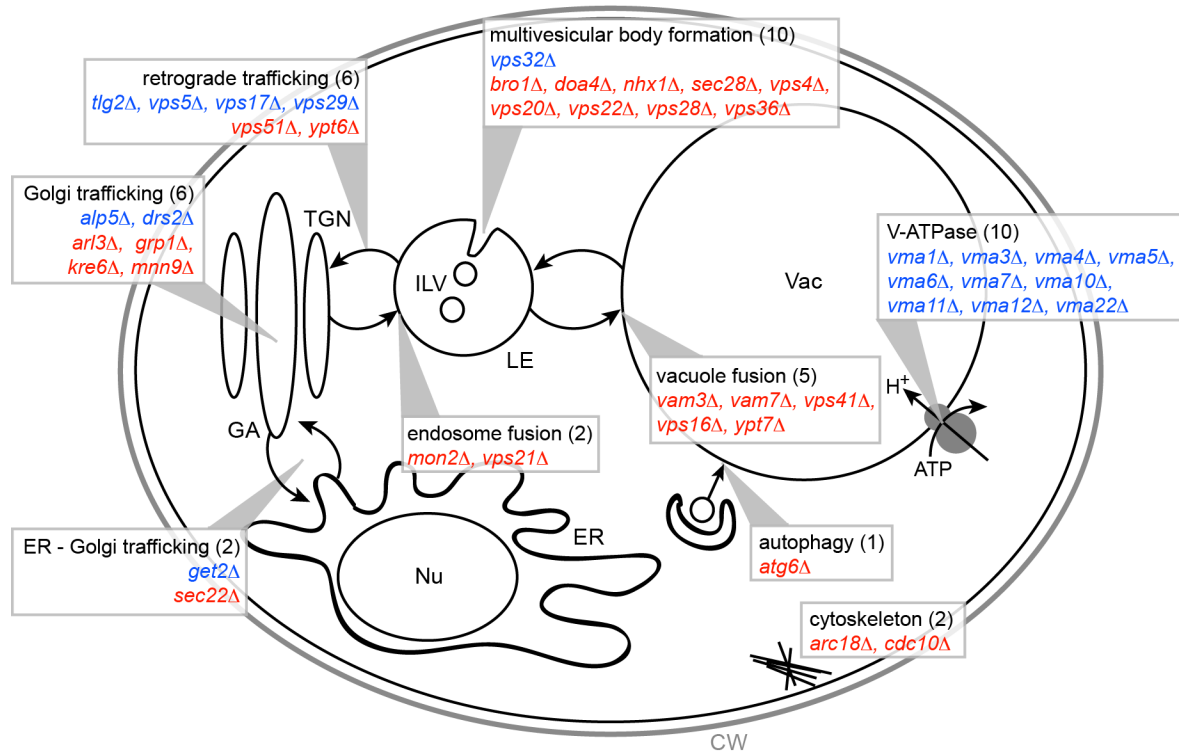

Supplement: Figure S2 — Vesicle trafficking defects lead to pHv dysregulation. Mutants with defective pHv also identified by genome-wide screens for endocytic trafficking defects and/or vacuole fusion defects are shown (numbers indicated in parentheses). Arrows indicate directional trafficking pathways between the following organelles: vacuole (Vac), late endosome (LE), intralumenal vesicles of multivesicular bodies (ILV), trans-Golgi network (TGN), Golgi apparatus (GA), endoplasmic reticulum (ER), nucleus (Nu), cytoplasmic (Cyto), plasma membrane (PM), and cell wall (CW); the V-ATPase, autophagic process and cytoskeleton are also shown. The mutants identified either showed basic (blue) or acidic vacuole (red). The pHv of mutants shown are listed in Supplementary Table S2. (PDF) [file pone.0017619.s002.pdf]
