# Supplementary material for: Genome-Wide Analysis Reveals the Vacuolar pH-Stat of Saccharomyces cerevisiae
Source: PLoS One. 2011 Mar 14;6(3):e17619. doi: 10.1371/journal.pone.0017619 (PMC3056714; doi:10.1371/journal.pone.0017619)
Supplement: Table S3 — Summary of major contributors to vacuolar pH-stat. Summary of 107 genes identified as contributors to the vacuole pH-stat under more than one external pH condition (see Figure 1d). The genes are sorted by effect of deletion on vacuole pH and by cellular function. (PDF) [file pone.0017619.s005.pdf]

Table S3. Gene deletions that cause defects in vacuole pH homeostasis in response to more than one external pH

ORF	gene name	cellular location	function
**A. Gene deletions that increase vacuole pH (n = 30)**			
V-type H^+-ATPase		vacuole, endocytic system	lumenal acidification
YDL185W	VMA1**	vacuole, endocytic system	subunit A of the peripheral catalytic V1 sector
YEL027W	VMA3	vacuole, endocytic system	subunit c of the integral membrane V0 sector
YOR332W	VMA4	vacuole, endocytic system	subunit E of V1
YKL080W	VMA5	vacuole, endocytic system	subunit C of V1
YGR020C	VMA7**	vacuole, endocytic system	subunit F of V1
YCL005W	VMA9	vacuole, endocytic system	subunit e of V0
YHR039C	VMA10	vacuole, endocytic system	subunit G of V1
YPL234C	VMA11	vacuole, endocytic system	subunit c' of V0
YKL119C	VMA12	endoplasmic reticulum	V-ATPase assembly
YHR060W	VMA22*	endoplasmic reticulum	V-ATPase assembly
YMR123W	PKR1**	endoplasmic reticulum	V-ATPase assembly
Sterol/lipid biosynthesis			
YGL012W	ERG4	endoplasmic reticulum	C-24(28) sterol reductase
YML008C	ERG6	endoplasmic reticulum	delta(24)-sterol C-methyltransferase
YGR037C	ACB1	cytoplasm, nucleus	acyl-CoA binding protein, ceramide biogenesis
Membrane trafficking			
YHR012W	VPS29	endosome	retromer complex, anterograde endocytic trafficking
YOR132W	VPS17	endosome	retromer complex, anterograde endocytic trafficking
YOR069W	VPS5*	endosome	retromer complex, anterograde endocytic trafficking
YPL195W	APL5	Golgi-vacuole vesicles	delta-subunit of the AP-3 coat complex, Golgi to vacuole membrane trafficking
YHR108W	GGA2	Golgi	gamma-adaptin ortholog, Golgi to endosome protein sorting
Post-translational protein modification			
YOR002W	ALG6	endoplasmic reticulum	alpha-1,3-glucosyltransferase, mutations in human orthologs are linked to congenital disorder of glycosylation type Ic
YPL227C	ALG5	endoplasmic reticulum	UDP-glucose:dolichyl-phosphate glucosyltransferase
YAL023C	PMT2	endoplasmic reticulum	protein O-mannosyltransferase
Cell signaling			
YPL176C	TRE1	plasma membrane	transferrin receptor-like, regulates the vacuole degradation of the metal transporter Smf1, an Nramp ortholog
YHR075C	PPE1	cytoplasm	phosphoprotein phosphatase 2A methyl esterase
YNL307C	MCK1/YPK1	cytoplasm	glycogen synthase kinase (GSK-3) ortholog
YDR195W	REF2	nucleus	mRNA/snoRNA processing, important for ion homeostasis
Other			
YLR131C	ACE2	cytoplasm, nucleus	transcription factor, activates expression of early G1 genes including CUP1, or metallothionein
YCL016C	DCC1	nucleus	sister chromatid cohesion
YOL072W	THP1	nucleus	nuclear pore-associated protein, mRNA export
**B. Gene deletions that decrease vacuole pH (n = 77)**			
F_1F_o ATP-synthase		mitochondrion	H^+-coupled ATP synthesis
YPL078C	ATP4	mitochondrion	b subunit of the stator stalk
YLR295C	ATP14	mitochondrion	h subunit of the F_o sector
YGR008C	STF2	mitochondrion	regulator of the F_1F0-ATP synthase
Sterol/lipid biosynthesis			
YGR007W	MUQ1	cytoplasm, nucleus	choline phosphate cytidylyltransferase, PE biosynthesis
YPL057C	SUR1/CSG1	intracellular	mannosylinositol phosphorylceramide (MIPC) synthase subunit, sphingolipid biosynthesis
YHL007C	STE20	cytoplasm, bud	PAK family kinase, negatively regulates sterol levels
Membrane trafficking			
YPL084W	BRO1	endosome	coordinates deubiquitination in the MVB pathway
YPR173C	VPS4	endosome, cytoplasm	AAA-type ATPase, catalyze dissassmbly of MVB protein Machinery
YPL002C	VPS22***	endosome	subunit of the ESCRT-II complex, protein sortin into lumen vesicles in the MVB pathway

YML001W	YPT7	vacuole	Rab-GTP binding protein, vacuole membrane fusion, trafficking and inheritance
YPL045W	VPS16***	vacuole, endosome	subunit of Vps-C complexes, vacuole fusion and endosome-vacuole trafficking
YIL076W	SEC28	Golgi-ER vesicles, endosome	epsilon-subunit of the AP-1 coat complex, Golgi-ER trafficking, mutant shows defective MVB formation
YPL051W	ARL3	Golgi	Ras GTPase, intra-Golgi vesicle trafficking
Post-translational protein modification			
YGL257C	MNT2	Golgi	mannosyltransferase
YPL053C	KTR6	membrane, Golgi?	Probable mannosylphosphate transferase, oligosaccharide biosynthesis
YJL184W	GON7	nucleus	EKC/KEOPS protein complex subunit, modification of N-linked oligosaccharides
YPL069C	BTS1	mitochondrion	geranylgeranyl diphosphate synthase, required for Rab-GTP binding protein function
Cell signaling			
YPL031C	PHO85	nucleus	cyclin-dependent kinase involved in global response to stress
YOR014W	RTS1	cytoplasm, nucleus	B-type regulatory subunit of protein phosphatase 2A
Transporters			
YFL011W	HXT10	plasma membrane	putative hexose transporter
YPL058C	PDR12	plasma membrane	ATP-binding cassette transporter, contributes to organic acid adaptation by transport
YPL060W	LPE10	mitochondrion	magnesium transporter, MIT superfamily
Protein degradation			
YPL003W	ULA1	unknown	protein degradation through neddylation
YGR003W	CUL3	cytoplasm, nucleus	cullin-based E3 ubiquitin-protein ligase, activated by neddylation for protein degradation
Transcriptional and translational regulation			
YPL064C	CWC27	spliceosome	pre-mRNA splicing
YPL029W	SUV3	mitochondrion	ATP-dependent RNA helicase, degradation of aberrant or unprocessed RNAs
YPR189W	SKI3	cytoplasm, nucleus	exosome mediated mRNA degradation, translation inhibition
YPL086C	ELP3	cytoplasm, nucleus	elongator complex subunit, chromatin remodeling
YPR179C	HDA3	nucleus	class II histone deacetylase complex subunit
YDR191W	HST4	nucleus, cytoplasm	Sir2-family NAD+-dependent protein deacetylase
YPL001W	HAT1	nucleus, cytoplasm	catalytic subunit of a histone acetyltransferase complex
YGL244W	RTF1	nucleus	RNA polymerase II-associated complex subunit, transcriptional regulation
YPL038W	MET31	cytoplasm, nucleus	zinc-finger DNA-binding protein, transcriptional regulation
YPL089C	RLM1***	nucleus	MADS-box transcription factor, activated by MAPK
YBR267W	REI1	cytoplasm, nucleus	pre-60S factor, ribosomal biogenesis
YPR132W	RPS23B	ribosome	ribosomal protein 28 of the 40S subunit, translational accuracy
YPR163C	TIF3***	ribosome	translation initiation factor eIF-4B, binds RNA
YPL037C	EGD1	cytoplasm, nucleus	beta-subunit of the nascent polypeptide-associated complex, protein targeting
YPL079W	RPL21B*	ribosome	component of the large 60S ribosomal subunt
YNL177C	MRPL22***	ribosome	component of the mitochondrial large ribosomal subunit
YKR006C	MRPL13	ribosome	component of the mitochondrial large ribosomal subunit
YLR312W-A	MRPL15	ribosome	component of the mitochondrial large ribosomal subunit
YPL013C	MRPS16	ribosome	component of the mitochondrial small ribosomal subunit
YPR166C	MRP2	ribosome	component of the mitochondrial small ribosomal subunit
Metabolism			
YPR167C	MET16	cytoplasm	3'-phosphoadenylsulfate reductase, methionine metabolism
YBR248C	HIS7	cytoplasm	glutamine amidotransferase, histidine biosynthesis
YPR145W	ASN1	cytoplasm	asparagines synthetase, asparagine biosynthesis
YER061C	CEM1	mitochondrion	beta-keto-acyl synthase, fatty acid synthesis
YEL029C	BUD16	cytoplasm, nucleus	putative pyridoxal kinase, vitamin B6 metabolism
YCR105W	ADH7	cytoplasm	cinnamyl alcohol dehydrogenase ortholog, fusel alcohol synthesis
YPL061W	ADH6	cytoplasm	aldehyde dehydrogenase, converts acetylaldehyde to acetate
Other			
YPL024W	RMI1	cytoplasm, nucleus	subunit of the RecQ helicase-Topo III complex
YPL059W	GRX5	mitochondrion	oxidoreductase involved in synthesis of iron-sulfur centers,

			electron transport
YPR200C	ARR2	unknown	arsenate reductase, converts arsenate to arsenite
Unknown function			
YDR068W	DOS2	cytoplasm	
YDR152W	GIR2	cytoplasm	highly-acidic RWD-domain containing protein
YDR525W-A [a]	SNA2	vesicles	two predicted TMDs, orthologous to PMP3 encoding a cation transporter
YDR540C	IRC4	cytoplasm, nucleus	
YFR012W [a]		membrane?	four predicted TMDs
YGR015C		mitochondrion?	
YGR021W		mitochondrion?	
YGL260W		unknown	neighbors PAU11
YGL261C	PAU11	membrane?	
YGL263W [a]	COS12	membrane?	two tandem repeats each with one predicted TMD
YMR073C	IRC21	cytoplasm	putative cytochrome oxidoreductase
YPL021W	ECM23	nucleus	negative regulator of transcription?
YPL030W	TRM44	cytoplasm	putative tRNA U44 2'-O-methyltransferase, neighbors PHO85
YPL054W	LEE1	unknown	zinc-finger protein
YPL055C	LGE1	nucleus	mutant forms abnormally large cells
YPL056C	LCL1	unknown	neighbors LGE1
YPL066W		cytoplasm, bud	
YPL071C		cytoplasm, nucleus?	located near BTS1
YPL077C		unknown	neighbors ATP4
YPR116W		mitochondrion	
YPR153W [a]		unknown	three predicted TMDs
YPR157W	TDA6	unknown	neighbors CUR1
YPR158W	CUR1	nucleus?	destabilizes [URE3] priors, BTN2 paralog

All gene deletions listed showed vacuole pH defects under more than one external pH.
* A second strain harboring an overlapping genomic deletion eliminated this ORF and also displayed this phenotype.
** Mutant did not grow under alkaline conditions.
*** Mutant did not grow under acidic conditions.
[a] Predicted membrane spans, potential transporter
